# Pattern of Maxillofacial Injuries and Determinants of Outcome in a Large Series of Patients admitted to a Level-I Trauma Center

**DOI:** 10.29252/beat-070214

**Published:** 2019-04

**Authors:** Mahnaz Yadollahi, Mojgan Behzadi Seyf Abad, Forough Pazhuheian

**Affiliations:** 1 *Trauma Research Center, Rajaee (Emtiaz) Trauma Hospital, Shiraz University of Medical Sciences, Shiraz, Iran *

**Keywords:** Maxillofacial trauma, Mortality, Injury Severity Score, Mechanism of injuries

## Abstract

**Objective:**

To investigate the severity of injuries and the pattern of jaw and facial injuries in trauma patients and also to determine the predictors of the outcome in these patients.

**Methods:**

This cross-sectional study was conducted on 2697 patients with facial trauma who referred to trauma center in Shahid Rajaee (Emtiaz) Hospital, Shiraz, Iran during 2010-2015. Injury severity score was determined through the conversion of injury codes of the International Classification of Diseases, tenth revision (ICD-10). Binary logistic regression by backward method was used to determine the partial effects of independent risk factors on death odds ratio.

**Results:**

The mean age of patients with maxillofacial injuries was 31.96 ± 15.80 years. The mean injury severity score (ISS) was 4.3 ± 4.4 and about 80% of the patients had an ISS between 1 and 8. Mandible fracture and ear injuries, respectively, were the most and the least prevalent types of maxillofacial injury. The odds ratio of death by motorcycle accident was 1.7 times higher than falling down in maxillofacial patients.

**Conclusion:**

Age, gender (male), ISS, and mechanism of injury were the significant predictors of mortality in the facial trauma patients. Mandible fracture and ear injury, respectively, were the most and the least prevalent types of maxillofacial injury. Our findings demonstrate the need for referral to the maxillofacial surgeon and maxillofacial surgery should be in connected with neurosurgical centers.

## Introduction

Facial injuries, in particular soft tissue injuries and fractures of the facial bones, are frequently occurring as a result of motor vehicle crashes, falls, violent assaults, and crashes during recreational activities such as bicycling and skiing [1]. Maxillofacial fractures may occur alone or in combination with other fractures. Fracture patterns may vary with mechanism of injury, magnitude and direction of impact force, and damage location [2].  From an anatomical point of view, the oral and maxillofacial area of the body is of vital and aesthetic importance and about one third of all injured patients have a type of injury in this area. More than 50% of patients surviving road traffic injuries  have at least one type of maxillofacial injury [3].  Moreover, the diagnosis of damages subsidiary to these injuries is important for oral and maxillofacial surgeons, since about 40% of patients with fractures in the middle part of the face are reported with an eye injury, of which about 30% have moderate to severe problems; moreover, in 17.5% of cases with facial fractures, there is a kind of closed brain injury and there are reports of cervical spine injuries with lower jaw fractures [4]. Face injuries may cause deformation or reduce the performance, for example, blindness or disturbance in jaw movement can be caused by a facial damage. Although facial injuries are rarely a threat to human life, facial trauma can also lead to death, as this trauma causes severe bleeding or breathing difficulty. The mentioned problems occasionally occur as isolated lesions, and they are more commonly associated with other serious injuries. Previous studies demonstrate that the rate of concomitant head injuries in cases with facial fracture is as high as 50-80%, depending on the site of the fracture. While intracranial injuries often occur in cases with frontal and maxilla bone fractures, they are less frequently associated with mandible lesions [5].

 A large proportion of traumatic injuries are associated with damages to the face and jaw. These injuries often vary in terms of the degrees of deformity and impairment that make treatment difficult. They can reduce the quality of life and the performance of the affected person [6]. In a research conducted during 1995-2000, it was found that after cardiovascular diseases, road accident are the second leading cause of death in Iran and most developing counties [7]. It has been reported that the cause of maxillofacial fractures is largely different in various countries. In countries such  as Jordan, Singapore, Nigeria, New Zealand, Denmark, and Japan motor vehicle crashes are the most common cause of maxillofacial fractures, while in Finland, the United States, and Sweden assault is reported as the most common etiological factor [8]. According to a report by the World Health Organization, global rate of mortality from road traffic injuries is 18.16 deaths per 100,000 that is lower than the rate observed in Iran by 32.14 deaths per 100,000 [9, 10]. Such epidemiological information can also be used to guide the future funding of public health programs toward prevention. To this end, independent investigators have conducted numerous studies on population groups from different countries, all with the common goal of elucidating the factors affecting maxillofacial injuries.

Understanding causes, severity, and distribution of facial trauma and the concomitant injuries can help to optimize the initial clinical treatment services and define the right time to involve oral surgeons. A clearer understanding of the demographic patterns of maxillofacial injuries will assist health care providers to plan and manage the treatment of traumatic maxillofacial injuries. The aim of the present study was to evaluate the frequency of maxillofacial injuries and risk factors affecting mortality in patients with maxillofacial injuries who referred to the trauma referral center in Shahid Rajaee (Emtiaz) Hospital, Shiraz, Iran during 2010-2015.

## Materials and Methods

This cross-sectional perspective study was conducted to evaluate the pattern and severity of maxillofacial injuries. To achieve this objective, data were collected from the records of 2697 patients with maxillofacial injuries who referred to trauma center in Shahid Rajaee Referral Hospital in Shiraz, Iran, between 2010 and 2015. All variables were collected from health information system(HIS) according to the preliminary checklist.  After a patient was screened and admitted, a unique eight-digit code called “SERIAL CODE” was generated by the hospital admission unit. Upon admission, the data on baseline demographic characteristics such as age, gender, time of admission, injury mechanism (motor-vehicle accident, pedestrian accident, assault, falling down, suicide, etc.), and type of maxillofacial injury such as eye injury, nose injury or fracture, skin scar, maxilla fracture, mandible fracture, ear injury, and multiple injuries (two or more type of injuries in face and jaw) were routinely recorded by the admissions unit. In addition, the injury severity score (ISS) (1-3, 4-8, 9-15, 16-24, >25) was determined based on the records. An ICD-10 code was determined for each patient based on primary and secondary diagnoses. Patients with multiple injuries were scored by adding the squares of the three highest AIS scores in three predetermined body regions. This process helped to achieve the ISS, which could range from 1 to 75. 

*Statistical analysis*

 Data were recorded using Microsoft Excel and transferred into the Stata 14 Statistical software. Statistical analysis was performed by Stata 14 and R3.4.2 was used for the visualization of the results. The patterns of maxillofacial injuries were classified into eight groups, including eye injuries, nose injures or fracture, lip & oral, skin scar, maxilla fracture, mandible fracture, ear injuries, and multiple injuries. Multiple logistic regression by backward method was used to determinate the partial effects of each independent variable including age, gender, mechanism of injury and injury severity score on the odds ratio of mortality as dependent variable from maxillofacial injuries. In addition, unconditional Logistic Regression Coefficients and Odds Ratios of most common mechanisms of injury were calculated to determine the predictors of Facial Trauma among trauma patients. A two-sided *P*-value of < 0.05 was considered to be statistically significant. 

## Results

The data were collected on a total number of 2697 patients with maxillofacial injuries. the mean age of 31.96±15.80 who referred to the hospital during 2010-2015. Of all, 2100 patients (99.48%) survived. More than half of the injured patients were in the age group of 18-30 years old. Maxillofacial injuries in males were 4 times more prevalent than that in females (2186 VS 511). Car (40.6%) and motorbike (24.2%) accidents were the main causes of maxillofacial injuries. The mean ISS of the patients with maxillofacial injuries was 4.3±4.4 and about 80% of the patients had an ISS between 1 and 8 (Table 1). 

**Table 1:** Descriptive statistics of the patients with maxillofacial trauma and other type of trauma

**Variables**	**With Face Trauma** **(n=2997)**	**Without Face Trauma** **(n=73253)**	***p*** **-value **
**Gender**			<0.001
Female	511(18.9)	18637(25.4)	
Male	2186(81.1)	54616(74.6)	
**Age**			<0.001
**Mean (SD) day**	32.7±15.4	34.6 ±16.6	
**Outcome**			0.033
Survived	2670(99.0)	72143(98.5)	
Died	27(1.0)	1110(1.5)	
**Mechanism of Injury**			<0.001
Car Accident	1094(40.6)	29026(39.6)	
Motorbike Accident	654(24.2)	14654(20.0)	
Pedestrian Accident	180(6.7)	5587(7.6)	
Assault	282(10.5)	7966(10.9)	
Falling Down	309(11.5)	11918(16.3)	
Struck by Object	175(6.5)	3943(5.4)	
Suicide	3(0.1)	159(0.2)	
**Injury Severity Score**			<0.001
**Mean (SD)**	4.3±4.4	5.6±5.3	
1-3	814(30.9)	17698(25.5)	
4-8	1444(54.8)	32990(47.5)	
9-15	278(10.6)	12892(18.6)	
16-24	66(2.5)	4007(5.8)	
>25	33(1.3)	1800(2.6)	
**Length of Stay**			<0001
**Mean (SD) day**	5.2±7.4	5.1±8.1	

**Fig. 1:**
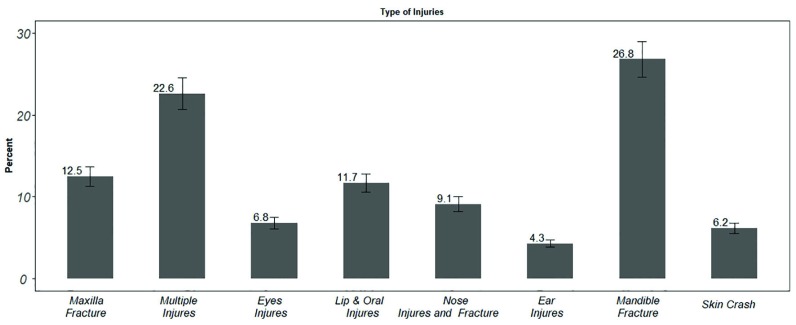
Type of maxillofacial injuries

Figure 1 presents the causes of maxillofacial injuries. As shown, mandible fracture and ear injuries, respectively, were the most and the least common types of maxillofacial injuries.  

**Fig. 2:**
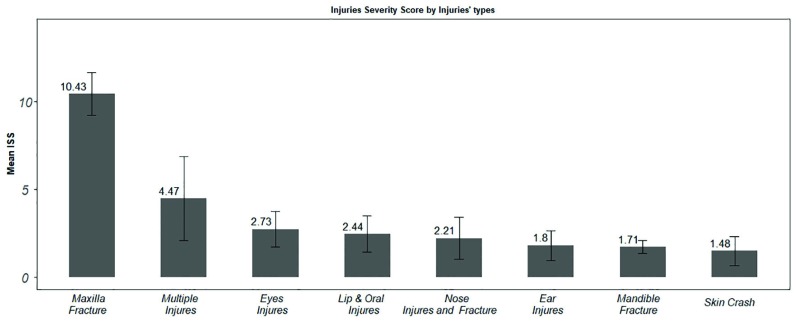
Mean injury severity score (ISS) of different types of maxillofacial injuries

 Figure 2 shows the severity of injury in any type of maxillofacial injuries. As shown, Maxilla fracture and skin scar had the highest and the lowest ISS . Moreover, the mean ISS in maxilla fractures was more than 8 times higher than that in mandible fractures (10.43 VA 1.71).

 Table 2 presents the contribution of different types of maxillofacial trauma in terms of the mechanism of injuries. As shown, car accident was the most common cause of injury in all types of maxillofacial trauma and the highest incidence rate. Skin scar and eye injuries were ranked as the second common mechanism of injury by assault. About 60% of all types of maxillofacial injuries were caused by car and motorcycle accident. Furthermore, car accident was identified as the main cause of lip and oral injuries in about 45% of all cases. In about 30% of the cases, car accident caused more than two injuries. Based on the results, suicide was the cause no type of maxillofacial injury. 

**Table 2:** Frequency of type of injuries by mechanism of injuries

Mechanism of injury	**Car Accident**	**Motorbike Accident**	**Pedestrian Accident**	**Assault**	**Falling Down**	**Struck by Object**	**Suicide**
**Type of injuries**	Frequency(%)						
**Eye injuries **	69(37.7)	32(17.5)	6(3.3)	39(21.3)	19(10.4)	18(9.8)	0
**Lip and oral injuries **	145(45.9)	79(25.5)	17(5.4)	24(7.6)	39(12.3)	12(3.8)	0
**Nose injuries and fracture**	114(46.5)	52(21.2)	16(6.5)	21(8.6)	32(13.4)	10(4.1)	0
**Skin scar **	64(38.3)	26(15.6)	6(3.6)	38(22.8)	23(13.8)	9(5.4)	1(0.6)
**Maxilla fracture**	138(40.8)	100(29.6)	24(7.1)	18(5.3)	33(9.8)	25(7.4)	0
**Mandible fracture**	304(42.1)	192(26.6)	42(5.8)	47(6.5)	75(10.4)	62(8.6)	0
**Ear injuries**	48(41)	22(18.8)	12(10.3)	20(17.1)	8(6.8)	7(6)	0
**Multiple Injuries**	212(34.8)	151(24.8)	57(9.4)	75(12.3)	80(13.1)	32(5.3)	2(0.3)

The obtained regression coefficient indicated that the odds ratio of death in males was 1.4 times higher than that in females. As the results of the logistics test showed, motorcycle accident and being struck by an object were the two most common causes of death, so that the odds ratio of death by motorcycle accident and being struck by an object was 1.7 times higher and the odds ratio of mortality by suicide was 0.38 times lower than the odds ratio of falling down that were significant. Odds ratio of death at the age of 65 and older was 1.07 (0.60-1.89) times more than the odds ratio of death at the age of 15- 44 years old. Aging increased the odds ratio of mortality, however, the increase was not significant (Table 3). 

**Table 3:** Logistic Regression Coefficients and odds ratios of the predictors of mortality among trauma patients

**Variables**	**Regression coefficient**	**Odds ratio**	**Standard Error**	***P*** **- value**	**95% CI for OR**
**Gender**					
Female	-	1	-	-	-
Male	.378	1.4	.050	<0.001	(1.3-1.6)
**Age**					
15-44	-	1	-	-	-
45-64	-.017	.98	.206	0.935	(0.65-1.47)
65>	.068	1.07	.291	0.815	(0.60-1.89)
**Mechanism of Injury**					
Falling Down	-	1	-	-	-
Motorbike Accident	.543	1.7	.070	<0.001	(1.5-1.9)
Pedestrian Accident	.217	1.2	.095	0.022	(1.0-1.4)
Car Accident	.374	1.4	.065	<0.001	(1.2-1.6)
Assault	.311	1.3	.084	<0.001	(1.1-1.6)
Struck by Object	.538	1.7	.096	<0.001	(1.4-2.0)
Suicide	-.318	0.72	.586	0.587	(0.21-2.2)
**Injury Severity Score **					
1-3	-	1	-	-	-
4-8	-.192	1.02	.073	.009	(0.7-0.9)
9-15	.258	1.29	.102	.012	(1.0-1.5)
16-24	.245	1.27	.159	.123	(0.9-1.7)
>25	.817	2.26	.296	.006	(1.2-4.0)

## Discussion

*Pattern of Maxillofacial Injuries*

  The results of our study revealed that mandible fracture and multiple injuries, respectively, accounted for 26.8% and 22.6% of all maxillofacial injuries. Numerous studies on trauma have investigated mandibular fracture epidemiology to determine effective prevention strategies, and establish accurate trauma evaluation protocols. According to a study by Canadian trauma center, 52% of the fractures occurred in individuals aged 21 to 40 years old, and 78% of the patients were male; in addition, there was a wide ethnic diversity between the patients [10]. Moreover, 60% of the patients had multiple mandibular fractures. 

In the present study, the most common type of damages due to car accidents were nose injuries and fracture (46.5%) and lip and oral injuries (45.9%). Thus, it can be concluded that car accidents followed by motorbike accidents are the most prevalent type of injuries among all the types of injuries. In addition,  falling down was identified as the main cause of nose injuries; it is in line with the results of a study by Salonen that showed the injury caused by fall-from-height was the most frequent cause of injury [11].  However, this findings are not consistent with the results of Max *et al*. study that reported falling dawn as the first cause of maxillofacial trauma [12].  The results of this study are also consistent with the results of Khatri’s study which reported that of all cases with injury 44% had face injury, 26% had nose injury, 14% had oral cavity injury, 10% had neck trauma, and 6% had ear injury [13]. In this study, age had no effect on facial trauma mortality. This finding is consistent with the results of numerous studies such as Norbega *et al*. study which reported the high prevalence of facial trauma among Brazilian patients affected by motorcycle accident [14]. The controversy in the findings can be attributed to differences in factors associated with traffic such as the number and types of vehicles . Overall, it can be concluded that the mechanism of injuries may differ by the characteristics of each country. 

*Mechanism of Injuries and Injury Severity Score*

 In the present study, road-traffic accidents, especially motorbike accidents, were identified as the leading risk factor for mortality from maxillofacial injuries (OR=1.4). Odds ratio of mortality from car and pedestrian accidents were 1.7 and 1.2, respectively. Yadollahi et al. showed that car accidents were the main mechanism of injuries and mortality in 39.6% of  all cases [15]. The results of a study in Saudi Arabia showed that the most frequently injured regions of the body were head and neck [16]. In the study by sadeghi bazargani et al. showed that 56% of total injuries related to head and face regions that most of damages were caused by car accident [17]. 

James Furness showed that the most frequently injured regions of the body with acute injury were shoulder (16.4%), ankle (14.6%), and head/face (13.3%), respectively. Their findings are not in line with our finding [18]. Rivara *et al*. conducted a case control study and showed that 34% of injured bicyclists had at least one type of facial injuries; they concluded that upper and mid-face injuries could be reduced by 65% by using helmets [19].

ISS is the most appropriate trauma score used for predicting mortality. The results of our study showed that the odds ratio of mortality was higher in patients with facial trauma. The results of this study are consistent with the results of a study by Thom Mayer et al. that reported ISS as an accurate predictor of both morbidity and mortality in all types of trauma such as face and neck injuries [20]. Although this study consistent with study by sherafati *et al*. that showed severity of crash and time to admission can predict mortality of patients [21]. As shown in Figure 2, Maxilla injuries had the highest ISS (with the mean score of 10.43), whereas this type of injury is ranked the third among the trauma injuries. In a study by K. E. Down it was shown that about 50% of patients had moderate to serve injuries but in this study the severity of injuries in 26% of patients with maxillofacial trauma was the same; it is due to the fact that facial trauma generally has a lower ISS than other type of injuries such as head and neck and abdomen injuries [22]. 

Moreover, Bagheri *et al*. showed a linear correlation between mortality and ISS among patients with facial trauma [23]. In a study by Max J.Scheyerer, it was reported that 59% of trauma patients needed maxillofacial surgeons and of all cases 72% had bleeding, 49% had chest injury, and 36% had subdual injury [11]. In our study, we assessed and compared the factors affecting mortality in terms of the mechanism of injuries and type of maxillofacial trauma; this comparison was one of the most significant strengths of this study. To the best of our knowledge, so far no study in Iran has been conducted on trauma and the risk factors of mortality in different injury groups, yet. Short post-trauma follow-up period was a limitation of our study. Due to the lack of data on other damaged body regions in patients, in addition to the jaw and face, we could not calculate and compare the risk of death due to the damages in such regions such as chest, head, neck, etc. According to the reviewed literature, this is the first study in Iran investigating death from jaw and facial injuries . For future research, it is suggested to consider competing mortality risk factors for all the types of maxillofacial injuries. Futurs studies are recommended to evaluate the effect of different factors such as seat belt and prevention of alcohol drinking on the decrease of maxillofacial traumas.

Maxillofacial injuries are often associated with a risk of other serious concomitant injuries, in particular traumatic brain injuries. Gender (being male), Injury Severity Score, and mechanism of injury were the significant predictors of mortality in the facial trauma patients. In addition, mandible fracture and ear injuries were the most and the least common types of maxillofacial injuries. Even though emergency operations are only necessary in rare cases, diagnosis and treatment of such concomitant injuries may be potentially overlooked or delayed in severely injured patients. Nevertheless, the need for immediate maxillofacial surgery is low. It seems necessary that treatment services for severely injured patients would be provided only by a limited number of major trauma centers, with a close collaboration between trauma, neuro, and maxillofacial surgeons.

## Conflict of Interest:

None declared.
